# Draft Genome Sequences of Marine Flavobacterium *Nonlabens* Strains NR17, NR24, NR27, NR32, NR33, and Ara13

**DOI:** 10.1128/genomeA.01165-14

**Published:** 2014-11-13

**Authors:** Masato Nakanishi, Pedro Meirelles, Ryohei Suzuki, Naoki Takatani, Sayaka Mino, Wataru Suda, Kenshiro Oshima, Masahira Hattori, Moriya Ohkuma, Masashi Hosokawa, Kazuo Miyashita, Fabiano L. Thompson, Ako Niwa, Toko Sawabe, Tomoo Sawabe

**Affiliations:** a Faculty of Fisheries Sciences, Hokkaido University, Hakodate, Japan; b Laboratory of Microbiology, Institute of Biology and Solid Action on Globalization and Environment, COPPE, Federal University of Rio de Janeiro (UFRJ), Rio de Janeiro, Brazil; c Center for Omics and Bioinformatics, Graduate School of Frontier Sciences, The University of Tokyo, Kashiwa, Japan; d Microbe Division/Japan Collection of Microorganisms, RIKEN BioResource Center, Ibaraki, Japan; e Department of Food and Nutrition, Hakodate Junior College, Hakodate, Japan

## Abstract

Here, we present the draft genome sequences of six carotenoid producers affiliated with *Nonlabens* spp. isolated from marine environments in both the northern and southern parts of Japan. The genomic information will help to elucidate the function and evolution of carotenoid synthetic gene clusters not only in the genus *Nonlabens* but also in the family *Flavobacteriaceae*.

## GENOME ANNOUNCEMENT

The etymology of “flavobacteria” originates from “yellow-bacteria” for the diverse array of bacteria showing Gram-negative, aerobic nonmotile yellow to orange colonies (1). The family *Flavobacteriaceae* was established for such bacterial groups and currently includes >114 genera (see http://www.bacterio.net/). One genus, *Nonlabens*, was proposed in 2005 for nonmotile orange nondiffusible pigment producers of a mesophilic estuarine origin (2). More recently, orange-pigmented flavobacteria were reclassified, in which *Persicivirga*, *Sandarakinotalea*, and *Stenothermobacter* were proposed to represent a single emended genus, *Nonlabens* (3). Ten species have been described in the genus *Nonlabens* today (2, 4–7). Ecophysiological and genomic studies revealed that *Nonlabens* strains possesses unique multiple proteorhodopsins (8, 9) and ulvan-degrading activity (10). However, carotenoid synthetic pathways that are attributed to orange colonies have not yet been fully elucidated. Strains NR17, NR24, NR27, NR32, and NR33 were isolated from a rock surface on the coast near Nanaehama, Hakodate, in the north of Japan. Strain Ara13 was isolated from a sandy beach on Ishigaki Island, Okinawa, in the south of Japan. Phylogenetic analyses on the basis of 16S rRNA gene sequences showed that five strains belong to *Nonlabens* spp.; in more detail, five strains (NR17, NR24, NR27, NR32, and NR33) were affiliated with *Nonlabens ulvanivorans* (11), and strain Ara13 was from *Nonlabens sediminis* (12), sharing 99.7 to 99.9% and 100% sequence similarities against the sequence of each type strain, respectively. A series of carotenoid identification analyses revealed the major carotenoid of these strains to be myxol.

The genome sequences of these *Nonlabens* spp. were sequenced with the Ion PGM system (Life Technologies, Carlsbad, CA) and assembled using Newbler version 2.8. The annotation and genome analysis were performed by Rapid Annotations using Subsystems Technology (RAST) (13). The sizes of the draft genome of *Nonlabens* strains NR17, NR24, NR27, NR32, NR33, and Ara13 are 3,252,740 bp, 3,216,957 bp, 3,258,856 bp, 3,260,523 bp, 3,091,813 bp, and 2,935,762 bp, comprise 42, 34, 50, 43, 25, and 22 contigs, and have G+C contents of 35.1%, 35.1%, 35.1%, 35.1%, 35.2%, and 35.3%, respectively. The number of redundancies are 25, 24, 24, 25, 23, and 48, and the *N*_50_ contig lengths are 362,590 bp, 233,893 bp, 235,448 bp, 322,501 bp, 449,344 bp, and 294,113 bp, respectively. The number of putative coding sequences (CDSs) are 3,683, 3,731, 3,686, 3,714, 3,543, and 2,952, the number of rRNA sequences are 3, 3, 3, 3, 2, and 3, and the number of tRNA sequences are 33, 32, 32, 33, 33, and 32 for strains NR17, NR24, NR27, NR32, NR33, and Ara13, respectively. Myxol synthetic gene clusters consisting of 5 core genes (*crtI*, *crtB*, *crtZ*, *crtD*, *crtYm*, and *crtA*) (14) are common genomic features among the newly isolated five *Nonlabens* strains. These strains have been deposited in the Japan Collection of Microorganisms as JCM 19296 (NR17), JCM 19314 (NR 24), JCM 19275 (NR27), JCM 19297 (NR32), JCM 19298 (NR33), and JCM 19294 (Ara13), respectively.

### Nucleotide sequence accession numbers.

The genome data have been deposited at DDBJ/EMBL/GenBank under the accession numbers BBLG01000001 to BBLG01000042, BBMM01000001 to BBMM01000034, BBNT01000001 to BBNT01000050, BBMJ01000001 to BBMJ01000043, BBMK01000001 to BBMK01000025, and BBML01000001 to BBML01000022 for *Nonlabens* strains NR17, NR24, NR27, NR32, NR33, and Ara13, respectively.

## References

[1] Holmes B Owen RJ McMeekin TA 1984 Genus *Flavobacterium* Bergey, Harrison, Breed, Hammer and Huntoon. 1923:97^AL^, p 353–361 *In* Krieg NR Holt JG (ed), Bergey’s manual of systematic bacteriology, vol 1 Williams & Wilkins, Baltimore, MD.

[2] Lau SCK Tsoi MMY Li X Plakhotnikova I Dobretsov S Wong P-K Pawlik JR Qian P-Y 2005 *Nonlabens tegetincola* gen. nov., sp. nov., a novel member of the family *Flavobacteriaceae* isolated from a microbial mat in a subtropical estuary. Int. J. Syst. Evol. Microbiol. 55:2279–2283. 10.1099/ijs.0.63810-0. 16280483

[3] Yi H Chun J 2012 Unification of the genera *Nonlabens*, *Persicivirga*, *Sandarakinotalea* and *Stenothermobacter* into a single emended genus, *Nonlabens*, and description of *Nonlabens agnitus* sp. nov. Syst. Appl. Microbiol. 35:150–155. 10.1016/j.syapm.2011.12.002. 22326813

[4] Lau SC Tsoi MMY Li X Plakhotnikova I Dobretsov S Wu M Wong P-K Pawlik JR Qian P-Y 2006 *Stenothermobacter spongiae* gen. nov., sp. nov., a novel member of the family *Flavobacteriaceae* isolated from a marine sponge in the Bahamas, and emended description of *Nonlabens tegetincola*. Int. J. Syst. Evol. Microbiol. 56:181–185. 10.1099/ijs.0.63908-0. 16403884

[5] Park S Yoshizawa S Chiura HX Muramatsu Y Nakagawa Y Kogure K Yokota A 2012 *Nonlabens marina* sp. nov., a novel member of the *Flavobacteriaceae* isolated from the Pacific Ocean. Antonie Van Leeuwenhoek 102:669–676. 10.1007/s10482-012-9765-4. 22736101

[6] Park S Kang C-H Yoon J-H 2013 *Nonlabens arenilitoris* sp. nov., a member of the family *Flavobacteriaceae* isolated from seashore sand. Antonie Van Leeuwenhoek 103:1125–1132. 10.1007/s10482-013-9893-5. 23423354

[7] Kwon YM Yang S-H Kwon KK Kim S-J 2014 *Nonlabens antarcticus* sp. nov., a psychrophilic bacterium isolated from glacier ice, and emended descriptions of *Nonlabens marinus* Park *et al*. 2012 and *Nonlabens agnitus* Yi and Chun 2012. Int. J. Syst. Evol. Microbiol. 64:400–405. 10.1099/ijs.0.056606-0. 24065773

[8] Kwon S-K Kim BK Song JY Kwak M-J Lee CH Yoon J-H Oh TK Kim JF 2013 Genomic makeup of the marine flavobacterium *Nonlabens* (*Donghaeana*) *dokdonensis* and identification of a novel class of rhodopsins. Genome Biol Evol. 5:187–199. 10.1093/gbe/evs134. 23292138 PMC3595038

[9] Yoshizawa S Kumagai Y Kim H Ogura Y Hayashi T Iwasaki W DeLong EF Kogure K 2014 Functional characterization of *Flavobacteria rhodopsins* reveals a unique class of light-driven chloride pump in bacteria. Proc. Natl. Acad. Sci. U. S. A. 111:6732–6737. 10.1073/pnas.1403051111. 24706784 PMC4020065

[10] Kopel M Helbert W Henrissat B Doniger T Banina E 2014 Draft genome sequence of *Nonlabens ulvanivorans*, an ulvan-degrading bacterium. Genome Announc. 2(4):e00793-14. 10.1128/genomeA.00793-14. 25125644 PMC4132620

[11] Barbeyron T Lerat Y Sassi JF Le Panse S Helbert W Collén PN 2011 *Persicivirga ulvanivorans* sp. nov., a marine member of the family *Flavobacteriaceae* that degrades ulvan from green algae. Int. J. Syst. Evol. Microbiol. 61:1899–1905. 10.1099/ijs.0.024489-0. 20833882

[12] Khan ST Nakagawa Y Harayama S 2006 *Sandarakinotalea sediminis* gen. nov., sp. nov., a novel member of the family *Flavobacteriaceae*. Int. J. Syst. Evol. Microbiol. 56:959–963. 10.1099/ijs.0.64055-0. 16627638

[13] Aziz RK Bartels D Best AA DeJongh M Disz T Edwards RA Formsma K Gerdes S Glass EM Kubal M Meyer F Olsen GJ Olson R Osterman AL Overbeek RA McNeil LK Paarmann D Paczian T Parrello B Pusch GD Reich C Stevens R Vassieva O Vonstein V Wilke A Zagnitko O 2008 The RAST server: Rapid Annotations using Subsystems Technology. BMC Genomics 9:75. 10.1186/1471-2164-9-75. 18261238 PMC2265698

[14] Teramotoa M Takaichi S Inomata Y Ikenaga H Misawa N 2003 Structural and functional analysis of a lycopene l-monocyclase gene isolated from a unique marine bacterium that produces myxol. FEBS Lett. 545:120–126. 10.1016/S0014-5793(03)00513-1. 12804761

